# Social Isolation in Early versus Late Adolescent Mice Is Associated with Persistent Behavioral Deficits That Can Be Improved by Neurosteroid-Based Treatment

**DOI:** 10.3389/fncel.2017.00208

**Published:** 2017-08-29

**Authors:** Andrea Locci, Philippe Geoffroy, Michel Miesch, Ayikoe-Guy Mensah-Nyagan, Graziano Pinna

**Affiliations:** ^1^The Psychiatric Institute, Department of Psychiatry, University of Illinois at Chicago, Chicago IL, United States; ^2^Laboratoire de Chimie Organique Synthétique, UMR 7177, Institut de Chimie de l’Université de Strasbourg Strasbourg, France; ^3^Biopathologie de la Myéline, Neuroprotection et Stratégies Thérapeutiques, INSERM U1119, Fédération de Médecine Translationnelle de Strasbourg (FMTS), Université de Strasbourg Strasbourg, France

**Keywords:** early stress, depression, PTSD, pharmacoresistance, allopregnanolone, GABA_A_ receptors, aggressive behavior, social isolation

## Abstract

Early trauma and stress exposure during a critical period of life may increase the risk of major depressive disorder (MDD) and post-traumatic stress disorder (PTSD) in adulthood. The first-choice treatment for MDD and PTSD are selective serotonin reuptake inhibitor (SSRI) antidepressants. Unfortunately, half of MDD and PTSD patients show resistance to the therapeutic effects of these drugs and more efficient treatments are essential. Both MDD and PTSD patients present reduced levels of allopregnanolone (Allo), a potent endogenous positive allosteric modulator of GABA action at GABA_A_ receptors which are normalized by SSRIs in treatment responders. Thus, Allo analogs or drugs that stimulate its levels may offer an alternative in treating SSRIs-non-responders. We tested several drugs on the aggressive behavior of early and late adolescent socially-isolated (SI) mice, a model of PTSD. Isolation in early adolescence (PND 21) induced more severe aggression than mice isolated at PND 45. A single non-sedating administration of *S*-fluoxetine (*S*-FLX; 0.375–1.5 mg/kg), or of the Allo analogs ganaxolone (GNX; 10 mg/kg), BR351 (1–5 mg/kg), or BR297 (0.3125–2.5 mg/kg), or of the endocannabinoid, *N*-palmitoylethanolamine (PEA; 5–20 mg/kg) all decreased aggression more effectively in late than early adolescent SI mice. Importantly, the number of drug non-responders was higher in early than late SI mice for all the drugs tested. The non-responder rate was more elevated (12–64%) after *S*-FLX treatment, while 100% of mice responded to a single administration of PEA at the dose range of 15–20 mg/kg. Moreover, GNX, BR351, and BR297’s antiaggressive effect persisted longer than *S*-FLX in both late and early SI mice. All drugs tested failed to alter locomotor activity of SI mice. Our results show that drugs that mimic Allo’s action or that induce Allo biosynthesis may be valuable for the treatment of “SSRIs non-responder” patients.

## Introduction

Exposure to traumatic experiences is associated with a drastic increase in the risk of developing psychiatric disorders, including major depressive disorder (MDD) and post-traumatic stress disorder (PTSD). These debilitating conditions affect 8–16% of the adult population in the United States and MDD alone is the most common neuropsychiatric disease worldwide (Kessler et al., 2005; Berton and Nestler, 2006; Whiteford et al., 2013).

Severe traumas, including abuse in women, child abuse and neglect, combat situations or sexual assault, result in a particularly serious form of chronic PTSD that is often comorbid with MDD and suicide (Prigerson et al., 2001), and is associated with a marked increase in vulnerability to substance and alcohol abuse as well as mood disorders such as bipolar disorder, generalized anxiety, and phobias (Famularo et al., 1992; Agid et al., 1999; Heim and Nemeroff, 2001; Kendler et al., 2004). Furthermore, a history of early-life trauma can predict a more severe and chronic depression and inadequate response to both pharmacological and psychotherapeutic treatments and even failure of treatment response in adulthood (Kessler, 1997; Zlotnick et al., 1997; Lanquillon et al., 2000; Wiersma et al., 2009; Shamseddeen et al., 2011; Nanni et al., 2012). For example, multiple childhood adverse experiences increased fourfold the risk of developing MDD during adult life (Felitti et al., 1998), and increased 2–5 times the risk of attempted suicide in childhood, adolescence, and adulthood (Dube et al., 2001). A study in women demonstrated a tight correlation between sexual or physical abuse in childhood and increased symptoms of anxiety, MDD, addiction and suicide in adulthood (McCauley et al., 1997). Of note, abuse in general but most notably, abuse occurring between 4 and 7 years of age predicted a lower response to 8 weeks of selective serotonin reuptake inhibitors (SSRIs) (Williams et al., 2016). SSRIs remain the most used antidepressants for decades, however, only 40–50% of MDD patients achieve remission, and more than 1/3 develop pharmacoresistance (Golden et al., 2002; Rush et al., 2006; Kemp et al., 2008). Likewise, for PTSD treatment, the only drugs approved by the FDA are the SSRIs sertraline and paroxetine but only 20% of SSRI-treated PTSD patients do not relapse (Westenberg, 1996; Walderhaug et al., 2010; Ipser and Stein, 2012). The reasons underlying SSRI-resistance can be multiple and can be found in genetic factors, pharmacokinetics, type of trauma, and comorbidity with other mental disorders (El-Hage et al., 2013; Willner et al., 2013). Failure to achieve full remission from MDD and PTSD symptoms in a large portion of patients indicates the need to develop alternative drugs for the treatment of non-responders.

Both MDD and PTSD are associated with altered GABAergic neurotransmission. For example, adolescent as well as adult MDD patients show a reduction of plasma, CSF, and cerebral cortex GABA concentrations (Luscher et al., 2011). Moreover, the expression of several GABA_A_ receptor subunits is altered in brain areas of MDD patients (Merali et al., 2004; Choudary et al., 2005; Klempan et al., 2009; Sequeira et al., 2009; Fatemi et al., 2013). Male Dutch veterans affected by PTSD show a significant reduction of benzodiazepine binding in cortex, hippocampus, and hypothalamus (Geuze et al., 2008), while male Viet Nam veterans show reduced binding in prefrontal cortex, Broadmann area 9 (Bremner et al., 2000). Furthermore, MDD and PTSD patients show low plasma, CSF, and brain levels of the GABA_A_ receptor-active, neurosteroid allopregnanolone (Allo) (Romeo et al., 1998; van Broekhoven and Verkes, 2003; Uzunova et al., 2006; Agis-Balboa et al., 2014). Depression during pregnancy and post-partum is likewise associated with changes in Allo levels (Nemeroff, 2008). Importantly, treatment with SSRIs normalizes CSF, plasma, and brain Allo levels in MDD patients, an effect associated with improved symptoms, while patients who fail to respond to SSRIs also fail to increase Allo levels (Romeo et al., 1998; Uzunova et al., 2006). Mouse stress models are probably the best translational approach to reproduce some of the behavioral and neurochemical alterations observed in MDD and PTSD patients. For example, the socially isolated (SI) mouse, a putative rodent model of PTSD, shows a time-dependent downregulation of corticolimbic Allo levels associated with behavioral dysfunction, such as aggressive behavior, anxiety-like behavior and altered contextual fear responses (Dong et al., 2001; Pinna et al., 2003; Pibiri et al., 2008; Nin et al., 2011a; Locci and Pinna, 2017a; Rasmusson et al., 2017). Furthermore, SI mice show changes in the expression of several GABA_A_ receptor subunits, which similar to PTSD patients, result in resistance to benzodiazepine’s pharmacological effects (Pinna et al., 2006b; Geuze et al., 2008; Pibiri et al., 2008; Nin et al., 2011b). Intriguingly, administration of low doses of SSRIs, acting as selective brain steroidogenic stimulants (SBSSs), normalize brain Allo levels and improve behavior in SI mice (Pinna et al., 2003, 2009). Likewise, administration of the Allo analog, ganaxolone (GNX), results in a dose-dependent improvement of emotional behavior (Pinna and Rasmusson, 2014).

In this paper, we hypothesize that early (PND 21) adolescence social isolation contributes to a more rapid and severe development of aggression and a lower pharmacological response to *S*-fluoxetine (*S*-FLX) than mice isolated in late adolescence (PND 45), which will be demonstrated by (i) a lower reduction in the rate of aggression, (ii) a lower duration of the drugs effect, and (iii) a higher percent of “non responders.” We also compare the pharmacological effect of *S*-FLX with that of neurosteroid-based treatments, including the endocannabinoid, *N*-palmitoylethanolamine (PEA) that stimulates brain Allo biosynthesis or the Allo analogs, GNX, BR351, and BR297 that directly act at GABA_A_ receptors.

The current study demonstrates that a single dose treatment with *S*-FLX, GNX, BR351, BR297, and PEA induced a stronger reduction of aggressive behavior in late than in early adolescent SI mice. Moreover, the rate of non-responders for all these drugs was higher in early SI mice and the pharmacological effect of these drugs was more enduring in late than early adolescent SI mice. Our data show that early SI mice develop earlier and more severe aggression than late SI mice and drugs like GNX, BR351, BR297, and PEA are stronger agents in counteracting these behavioral deficits.

## Materials and Methods

### Animals

Male Swiss–Webster mice (Harlan Breeders) (18–30 g body weight), maintained under a 12-h dark/light cycle with food and water ad libitum were used for all experiments. Mice were housed individually in a cage of dimensions 24 cm × 17 cm × 12 cm. For our experiments, we used two mice experimental groups for the study of drug effect in different age conditions. The first group was isolated at 21 days (“early adolescent SI mice,” PND 21), while the second group at 45 days (“late adolescent SI mice,” PND 45). The animals were exposed to behavioral testing after 6 weeks of isolation. The vivarium temperature was kept at 24°C and the humidity near 65%. All experiment protocols were approved by the Office of Animal Care and Institutional Biosafety Committee and the Office of the Vice Chancellor for Research of the University of Illinois at Chicago.

### Drug Treatments

*S*-fluoxetine (*S*-FLX) (0.375–1.5 mg/kg) was obtained from Eli Lilly and Company (Indianapolis, IN, United States). GNX (10 mg/kg; EC_50_ dose, previously established in Pinna and Rasmusson, 2014) was obtained from Marinus Pharmaceuticals, Inc. (Boston, MA, United States). BR351 (1–5 mg/kg) and BR297 (0.3125–2.5 mg/kg) were obtained from NeuroRhine Consortium (Strasbourgh, France). *N*-palmitoylethanolamine (PEA; 5–20 mg/kg) was purchased from Epitech Group Research lab (Saccolongo, Italy). All the drugs were dissolved in saline solution containing 0.5% Tween-80 (Sigma Aldrich, St. Louis, MO, United States), and were injected intraperitoneally (i.p.), 60 min before behavioral tests.

### Resident–Intruder Test

To test aggression, a male intruder mouse of the same strain as the resident mouse, was placed in a resident home cage (24 cm × 17 cm × 12 cm) and resident–intruder interactions were videotaped for 10 min. Aggressive behavior of SI mice was characterized by an initial pattern of exploratory activity around the intruder, followed by rearing and tail rattle, accompanied within a few seconds by wrestling and/or a violent biting attacks. The total number of wrestling and attacks during the 10 min observation period was measured as previously described (Pinna et al., 2003, 2005), 60 min after drug administration. For every drug studied, we first exposed SI mice to three behavioral sessions, and then we calculated the average of basal aggression level for every single SI mouse; 2 days later, we tested the aggression levels following treatment. Treated animals showing a reduction of the number of attacks less than 30% vs. the respective basal control values were considered as “low- to non-responders.”

### Locomotor Activity

A computerized AccuScan 12 Animal Activity Monitoring System (Columbus Instruments, Columbus, OH, United States) assisted by VERSAMAX software (AccuScan Instruments, Columbus, OH, United States) was used to quantify locomotor activity (Pinna et al., 1997, 2006a). Each activity cage consisted of a 20 cm × 20 cm × 20 cm Perspex box surrounded by horizontal and vertical infrared sensor beams. Horizontal sensors’ beam interruptions were taken as a measure of horizontal activity. Activity was recorded from SI mice for 10 min beginning 60 min after a single injection of the drug.

### Statistical Analyses

Results are presented as means ± SEMs unless otherwise indicated. Student’s *t*-test, one-way ANOVA and two-way ANOVA repeated measures followed by Bonferroni *post hoc* test were performed to analyze experimental data; significance was set at *p* < 0.05. EC_50_ values were calculated from dose-response curves analyzed by the “quantal dose-response: probits test” using the computer program of Tallarida and Murray equipped with a statistical package (Tallarida and Murray, 1987). Statistical comparisons among the different EC_50_s were performed with the “cohort package software.”^1^

## Results

### Development of Aggressive Behavior in Late and Early SI Adolescent Mice

**FIGURE 1:**
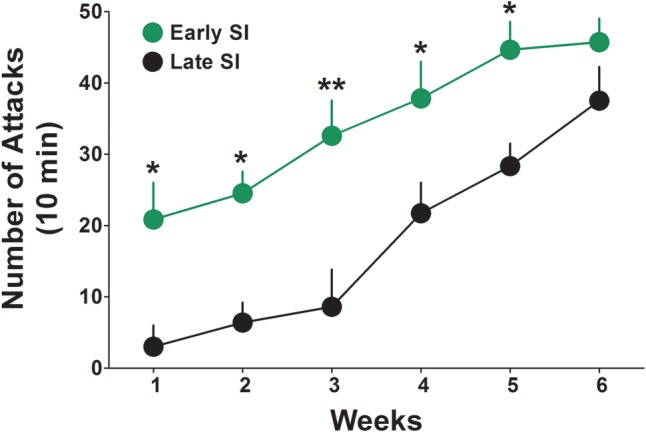
Onset of aggressive behavior in early and late adolescent SI mice. Representation of the development of the aggressive behavior in early (green) and late (black) adolescent SI mice from week 1 to 6 of social isolation. Early adolescent social isolation (started at day 21 of life, PND 21) induced a more severe development of aggression compared to late adolescent isolation (started at day 45 of life, PND 45). Data represent the mean ± SEM of 15 mice. ^∗^*p* < 0.05 and ^∗∗^*p* < 0.001, when compared with late adolescent SI mice at the same time point.

The basal levels of aggressive behavior were determined in both late and early SI mice starting from the first week of isolation, by testing resident–intruder interactions once a week for 6 weeks (**Figure 1**). We found that early isolation, which was started at PND 21, induced a more rapid and severe development of aggression compared to late social isolation, which was started at PND 45. Two-way ANOVA repeated measures revealed a significant effect of “onset of isolation” [*F*(1,140) = 42.23; *p* < 0.0001], an effect of “time of test” [*F*(5,140) = 16.67; *p* < 0.0001], but no interaction between factors [*F*(5,140) = 0.763; *p* = 0.578]. Bonferroni *post hoc* test showed a significant difference in the aggressive behavior between late and early SI mice in every week tested except week 6 (**Figure 1**). Aggression levels were studied only in SI mice given that group-housed conditions do not account for relevant levels of aggression (Pinna et al., 2003, 2005).

### *S*-FLX More Potently Improves Aggression in Late Than Early SI Mice

**FIGURE 2:**
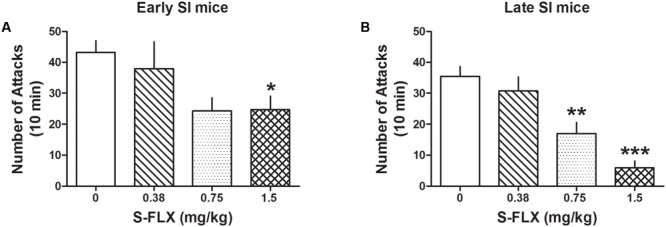
*S*-fluoxetine (*S*-FLX) decreases aggressive behavior in early and late adolescent SI mice. *S*-FLX at the doses of 0.375, 0.75, and 1.5 mg/kg, i.p., was administered both to early **(A)** and late **(B)** adolescent SI mice 60 min before the resident–intruder test. In late adolescent SI mice, *S*-FLX reduced aggression at the dose of 0.75 mg/kg with an EC_50_ dose of 0.85 mg/kg, while in early adolescent SI mice, *S*-FLX induced a decrease of aggression with an EC_50_ dose of >1.5 mg/kg. Data represent the mean ± SEM of 13–18 mice. ^∗^*p* < 0.05; ^∗∗^*p* < 0.01; ^∗∗∗^*p* < 0.001, when compared with basal control levels of aggression.

Administration of several doses of the SSRI, *S*-FLX (0.375, 0.75, and 1.5 mg/kg, i.p.) resulted in a stronger dose-dependent decrease of aggression in late than in early SI resident mice toward a same-sex intruder. One-way ANOVA showed that *S*-FLX reduced aggression in both late [*F*(3,84) = 13.08; *p* < 0.0001] and early SI mice [*F*(3,91) = 3.823; *p* = 0.013] (**Figure 2**). Bonferroni *post hoc* test showed a significant reduction in the number of attacks at the dose of 0.75 mg/kg (-52%, *p* < 0.01, *n* = 13) as well as 1.5 mg/kg (-83%, *p* < 0.001, *n* = 17) in late SI mice (EC_50_ dose = 0.85 mg/kg), but only at the highest dose (1.5 mg/kg) in early SI mice (-43% vs. basal value, *p* < 0.05, *n* = 18) (EC_50_ dose > 1.5 mg/kg). The *S*-FLX dose of 1.5 mg/kg was more potent in late [+316%, *t*(33) = 3.761, *p* = 0.0007] than early SI mice.

### GNX, BR351, and BR297 Decrease Aggression in Early and Late SI Mice

**FIGURE 3:**
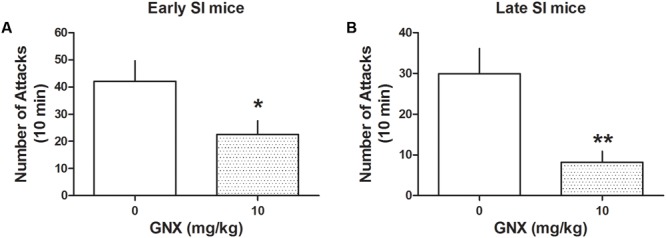
The analog of Allo, ganaxolone (GNX) ameliorates aggressive behavior in early and late adolescent SI mice. GNX (10 mg/kg, EC_50_ dose) was administered both to early **(A)** and late **(B)** adolescent SI mice 60 min before the exposure to a resident–intruder test. Equal doses of GNX showed a more powerful improvement in social isolation-induced aggression of late adolescent SI mice. Data represent the mean ± SEM of 12–13 mice. ^∗^*p* < 0.05; ^∗∗^*p* < 0.01, when compared with basal control levels of aggression.

Administration of GNX at the EC_50_ dose (10 mg/kg, i.p.; previously established in Pinna and Rasmusson, 2014) decreased aggression both in early and late SI mice [PND 21: -72%, *t*(24) = 3.208, *p* = 0.0038, *n* = 13; PND 45: -46%, *t*(28) = 2.164, *p* = 0.039, *n* = 12] (**Figure 3**). The effect of GNX was stronger in late compared with early SI mice [+197%, *t*(22) = 2.389, *p* = 0.026].

**FIGURE 4:**
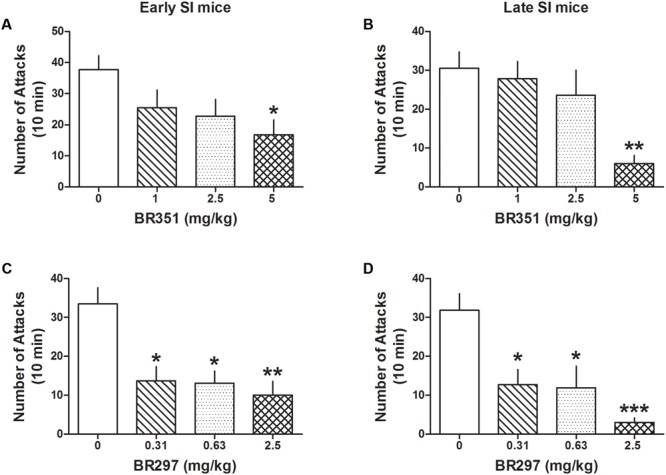
The analog of Allo, BR351 and BR297 decrease aggressive behavior in early and late adolescent SI mice. BR351 at the doses of 1, 2.5, and 5 mg/kg was administered both to early **(A)** and late **(B)** adolescent SI mice 60 min before the exposure to a resident–intruder test. Only the dose of 5 mg/kg improved aggression of SI mice with higher potency in late (EC_50_ = 3.75 mg/kg) than early (EC_50_ = 4.5 mg/kg) adolescent SI mice. BR297 at the doses of 0.3125, 0.625, and 2.5 mg/kg was administered both to early **(C)** and late **(D)** adolescent SI mice 60 min before the exposure to a resident–intruder test. Dose-response data analyses showed that BR297 was equally potent in decreasing aggression of early and late adolescent SI mice with an EC_50_ dose of 0.25 mg/kg. Data represent the mean ± SEM of 10–16 mice. ^∗^*p* < 0.05; ^∗∗^*p* < 0.01; ^∗∗∗^*p* < 0.001, when compared with basal control levels of aggression.

BR351 (1, 2.5, and 5 mg/kg) reduced aggression in late [*F*(3,62) = 4.458; *p* = 0.0067] and early SI mice [*F*(3,70) = 3.5303; *p* = 0.0192] (**Figures 4A,B**) only at the highest dose. Bonferroni analyses showed a reduction of attacks in late SI mice at the dose of 5 mg/kg (-80% vs. basal value, *p* < 0.01, *n* = 12) (EC_50_ dose = 3.75 mg/kg), and in the early SI mice at the dose of 5 mg/kg (-56%, *p* < 0.05, *n* = 16) (EC_50_ dose = 4.5 mg/kg). There was only a trend showing a higher potency of BR351 (5 mg/kg) in late compared to early SI mice [+179%, *t*(26) = 1.821, *p* = 0.0801].

BR297 (0.3125, 0.625, and 2.5 mg/kg) robustly reduced aggression in late [*F*(3,62) = 8.213; *p* < 0.0001] and early SI mice [*F*(3,74) = 7.512; *p* = 0.0002] (**Figures 4C,D**). Bonferroni showed a reduction of aggression at all the doses tested in the late (0.3125 mg/kg: -60%, *p* < 0.05, *n* = 11; 0.625 mg/kg: -63%, *p* < 0.05, *n* = 11; 2.5 mg/kg: -91%, *p* < 0.001, *n* = 12) (EC_50_ dose = 0.25 mg/kg) and in early SI mice (0.3125 mg/kg: -59%, *p* < 0.05, *n* = 10; 0.625 mg/kg: -61%, *p* < 0.05, *n* = 14; 2.5 mg/kg: -70%, *p* < 0.01, *n* = 16) (EC_50_ dose = 0.25 mg/kg). The effect of BR297 (2.5 mg/kg) showed a trend to lower potency in the early SI mice [+235%, *t*(26) = 1.66, *p* = 0.108].

### PEA Inhibits Aggression in Early and Late SI Mice

**FIGURE 5:**
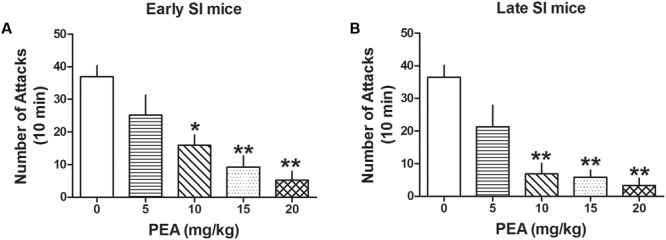
The endocannabinoid PEA strongly decreases aggressive behavior in early and late SI mice. PEA at the doses of 5, 10, 15, and 20 mg/kg was administered both to early **(A)** and late **(B)** SI mice 60 min before the exposure to a resident–intruder test. The EC_50_ values were 8 and 6.5 mg/kg, respectively. Data represent the mean ± SEM of 7–17 mice. ^∗^*p* < 0.01; ^∗∗^*p* < 0.001, when compared with basal control levels of aggression.

Administration of PEA (5, 10, 15, and 20 mg/kg, i.p.) inhibited aggression both in late [*F*(4,44) = 14.081; *p* < 0.0001] and early SI mice [*F*(4,98) = 12.327; *p* < 0.0001] (**Figure 5**). Bonferroni *post hoc* analysis showed a significant reduction of the number of attacks in the late (EC_50_ dose = 6.5 mg/kg) as well as early SI mice (EC_50_ dose = 8 mg/kg) at the doses of 10 mg/kg (-81%, *p* < 0.001, *n* = 7 and -57%, *p* < 0.01, *n* = 15, respectively), 15 mg/kg (-84%, *p* < 0.001, *n* = 7 and -75%, *p* < 0.001, *n* = 13, respectively) and 20 mg/kg (-91%, *p* < 0.001, *n* = 7 and -86%, *p* < 0.001, *n* = 17, respectively). Furthermore, our findings suggest no statistical differences in the effect of PEA among early and late adolescent SI mice.

### Duration of Drug-Induced Anti-aggressive Effects in Early and Late SI Adolescent Mice

**FIGURE 6:**
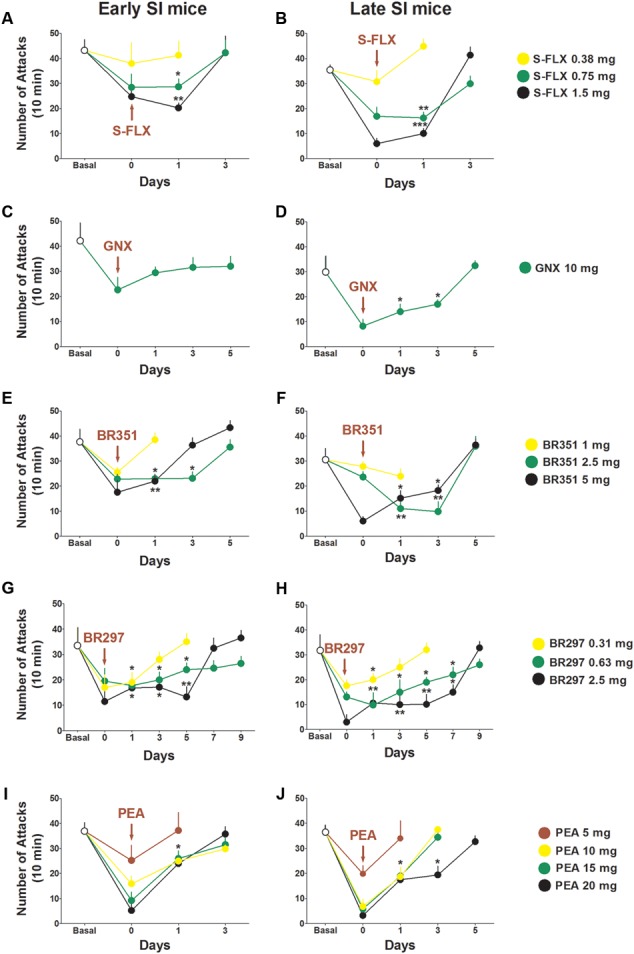
Drug-induced time-dependent anti-aggressive effect in late and early adolescent SI mice. Graphs represent the gradual reinstatement of aggressive behavior both in early **(A)** and late **(B)** adolescent SI mice during the period following the single dose administration of *S*-FLX (Day 0). Aggression rapidly rebounded after 3 days from *S*-FLX administration in both groups of mice. After an EC_50_ dose of GNX aggressive behavior rebounded to basal values only after 1 day in early adolescent **(C)** SI mice and after 5 days in late adolescent **(D)** SI mice. The gradual time-dependent reinstatement of aggressive behavior lasted 5 days in early **(E)** and late **(F)** adolescent SI mice after the administration of BR351 at the dose of 2.5 mg/kg; the duration of BR351 effect was 5 days also at the dose of 5 mg/kg in late SI mice, and 3 days in early SI mice. The anti-aggressive effect of a single dose administration of BR297 was long-lasting and required 7 days in early SI mice **(G)**, and 9 days in late SI mice **(H)** to return to basal values. Aggressive behavior was reinstated after 3 days following a single dose administration with PEA in early SI mice **(I)** at the doses of 10, 15, and 20 mg/kg, as well as in late SI mice **(J)** at the doses of 10 and 15 mg/kg. The effect of PEA at the dose of 20 mg/kg was extinguished after 5 days in late SI mice **(J)**. Data represent the mean ± SEM of 7–18 mice. ^∗^*p* < 0.05; ^∗∗^*p* < 0.01, when compared with basal control levels of aggression.

The duration of the anti-aggressive effect for each drug was assessed in a follow-up study after a single dose administration (**Figure 6**). Aggression rapidly rebounded after 1 day of *S*-FLX (0.375 mg/kg) administration and after 3 days at the dose of 0.75 mg/kg [PND 21, day 1: *t*(62) = 2.087, *p* = 0.041; PND 45, day 1: *t*(58) = 3.444, *p* = 0.0011] and 1.5 mg/kg [PND 21, day 1: *t*(63) = 3.122, *p* = 0.0027; PND 45, day 1: *t*(59) = 3.59, *p* = 0.0007] both in early and late adolescent SI mice.

GNX anti-aggressive effect (10 mg/kg) lasted 5 days in late SI mice [day 1: *t*(27) = 2.408, *p* = 0.0232; day 3: *t*(27) = 2.069, *p* = 0.0482]; the same trend was observed in early SI mice without significant effect.

Aggression was restored 1 day after BR351 at the dose of 1 mg/kg, and after 5 days at the doses of 2.5 mg/kg [day 1: *t*(51) = 2.979, *p* = 0.0044; day 3: *t*(47) = 3.034, *p* = 0.0039] and 5 mg/kg [day 1: *t*(47) = 2.263, *p* = 0.0283; PND 45, day 3: *t*(48) = 2.083, *p* = 0.0426] in late SI mice. Moreover, BR351 anti-aggressive effect rapidly extinguished at the dose of 1 mg/kg, after 5 days at the dose of 2.5 mg/kg [day 1: *t*(42) = 2.299, *p* = 0.0266; day 3: *t*(42) = 2.038, *p* = 0.0479], and after 3 days at the dose of 5 mg/kg [day 1: *t*(42) = 2.188, *p* = 0.0343].

BR297 at 0.625 mg/kg [day 1: *t*(46) = 3.395, *p* = 0.0014; day 3: *t*(41) = 2.195, *p* = 0.0339; day 5: *t*(42) = 2.269, *p* = 0.0285; day 7: *t*(47) = 2.413, *p* = 0.0198] and 1.5 mg/kg doses [day 1: *t*(46) = 3.320, *p* = 0.0018; day 3: *t*(47) = 3.441, *p* = 0.0012; day 5: *t*(46) = 3.552, *p* = 0.0016; day 7: *t*(46) = 2.511, *p* = 0.0156] inhibited aggression for 9 days in late SI mice. Furthermore, BR297 dose of 0.3125 mg/kg significantly reduced aggressive behavior for 3 days in the same experimental group [day 1: *t*(50) = 2.154, *p* = 0.0361]. The long-term effect of BR297 in early SI mice was similar to that observed in late SI mice but not identical. In fact, the duration of the effect was 3 days at the dose of 0.3125 mg/kg [day 1: *t*(52) = 2.175, *p* = 0.0342], and 7 days at the dose of 0.625 mg/kg [day 1: *t*(52) = 2.234, *p* = 0.0298; day 3: *t*(55) = 2.083, *p* = 0.0419; day 5: *t*(58) = 2.038, *p* = 0.0461] and 2.5 mg/kg [day 1: *t*(53) = 2.518, *p* = 0.0149; day 3: *t*(53) = 2.386, *p* = 0.0206; day 5: *t*(53) = 3.159, *p* = 0.0029].

Finally, PEA anti-aggressive effect lasted less than 3 days in late SI adolescent mice at the dose of 10 mg/kg [day 1: *t*(26) = 2.551, *p* = 0.017] and 15 mg/kg [day 1: *t*(26) = 2.309, *p* = 0.0291]. The anti-aggressive effect persisted for 3 days only in late adolescent SI mice after administration of PEA at the dose of 20 mg/kg [day 1: *t*(26) = 2.656, *p* = 0.0133; day 3: *t*(26) = 2.258, *p* = 0.0326]. Even though a similar trend was found in early adolescent SI mice, a significant effect was observed only at the dose of 20 mg/kg [day 1: *t*(60) = 2.044, *p* = 0.0453].

### Drug Non-response Rate in Early and Late SI Mice

The rate of SI mice that did not respond to the drugs’ pharmacological action was assessed by a decrease of aggression of less than or equal to 30%. Generally, a higher rate of non-response was assessed in early vs. late SI mice (see **Table 1**, for details). The percentage of non-responders to *S*-FLX at the higher dose of 1.5 mg/kg was 11.64 and 22.22% in late and early SI mice, respectively. Both in the late and early SI mice, the non-response rate at the highest doses tested of BR351 (5 mg/kg) was 8.33 and 13.33%, respectively, and at the BR297 dose of 2.5 mg/kg; 0 and 6.25%, respectively. Importantly, all early SI mice responded to PEA at the doses of 15 and 20 mg/kg; in addition, all late SI mice showed an inhibition of aggression at the PEA doses of 10, 15, and 20 mg/kg. Finally, the non-response rate at the EC_50_ dose of GNX (10 mg/kg) for both late and early SI mice was 15.38 and 25%, respectively.

**Table 1:** Rate of adolescent SI mice that show resistance to the single administration of *S*-fluoxetine (*S*-FLX), ganaxolone (GNX), BR351, BR297, and PEA.

Drug treatment	“Non-responders” Late SI mice (%)	“Non-responders” Late SI mice (n)	“Non-responders” Early SI mice (%)	“Non-responders” Early SI mice (n)
*S*-FLX 0.375 mg *S*-FLX 0.75 mg *S*-FLX 1.5 mg GNX 10 mg BR351 1 mg BR351 2.5 mg BR351 5 mg BR297 0.3125 mg BR297 0.625 mg BR297 2.5 mg PEA 5 mg PEA 10 mg PEA 15 mg PEA 20 mg	64.3% 23.1% 11.8% 15.4% 54.6% 40.0% 8.3% 18.2% 18.2% 0% 42.9% 0% 0% 0%	9/14 3/13 2/17 2/13 6/11 4/10 1/12 2/11 2/11 0/12 3/7 0/7 0/7 0/7	64.3% 53.3% 22.2% 25.0% 50.0% 20.0% 13.3% 30.0% 21.4% 6.3% 53.9% 26.7% 0% 0%	9/14 8/15 4/18 3/12 8/16 3/15 1/16 3/10 3/14 1/16 7/13 4/15 0/13 0/17

### Effects of Different Drug Treatments on Locomotor Activity in Early and Late SI Mice

**FIGURE 7:**
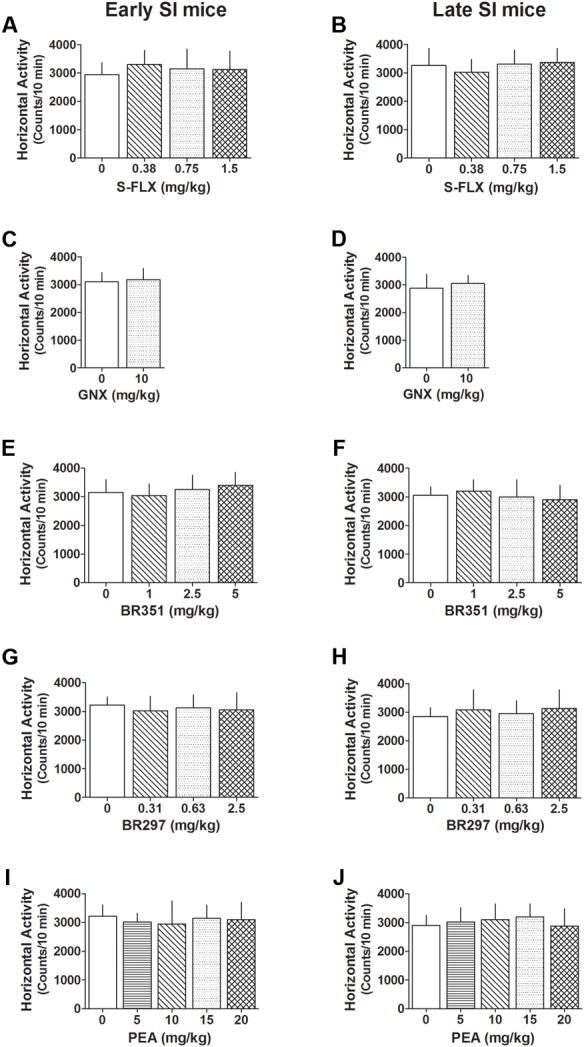
Effect of different drug treatments on locomotor activity both in late and early adolescent SI mice. *S*-FLX **(A,B)**, GNX **(C,D)**, BR351 **(E,F)**, BR297 **(G,H)**, and PEA **(I,J)** were administered to early (left side) and late (right side) adolescent SI mice 60 min before the exposure to the locomotor activity test. All drugs at the dose tested failed to alter locomotion patterns of SI mice. Data represent the mean ± SEM of 7–18 mice.

A summary of the locomotor activity after all drug tested is reported in **Figure 7**. *S*-FLX did not reduce exploratory activity in late and early SI mice at all doses tested. Similarly, exploratory activity was not altered by a single dose treatment with BR315, BR297, GNX as well as PEA at all doses tested.

## Discussion

In this study, we focused on the aggression expressed by SI mice because it is easy to reproduce, reliable to measure, and fails to decrease after multiple tests (Pinna et al., 2003, 2004; Matsumoto et al., 2005; Nelson and Pinna, 2011). Basal levels of aggression were determined in three resident–intruder tests before performing each of the drug-treated aggression experiment during which, aggression levels for each mouse were monitored before, during and after drug treatment. We compared the effects induced by different classes of drugs, i.e., SSRIs at steroidogenic doses that act as SBSSs, neurosteroid analogs, and endocannabinoids that induce neurosteroidogenesis, on the aggression of mice socially isolated in early (PND 21) and late (PND 45) adolescence. Our findings show that: (1) social isolation in early adolescence results in faster development of and a more persistent aggression than isolation in late adolescence (**Figure 1**); (2) early isolation was associated with a higher treatment resistance rate (**Table 1**); and (3) a lower duration of the drugs’ anti-aggressive effects (**Figure 6**). A single dose of the Allo novel analog BR297 (**Figure 4**), and the endocannabinoid, PEA (**Figure 5**) induced a dose-dependent robust anti-aggressive effect and the anti-aggressive effect of BR297 was long-lasting. These effects were compared to *S*-FLX’s potency, duration of effects and non-response rate. BR297 and PEA appeared to be more efficacious as anti-aggressive agents than *S*-FLX in both early and late adolescent isolation. Collectively, these results are suggestive of the clinical findings observed in subjects who were exposed to early life traumas and have received treatment for depression (Williams et al., 2016). First, mice isolated at PND21 developed a more severe and persistent aggressive behavior when compared to those isolated at PND45; second, this effect is associated with a higher “non-response” rate toward the drugs tested and a weaker response to *S*-FLX; third, there was a reduced temporal improvement of behavior following a drug’s single dose administration.

One of the most well-characterized behavioral dysfunctions following protracted social isolation in rodents is the development of aggressive behavior (Valzelli, 1969; Pinna et al., 2003). In SI mice, aggression co-occurs with other emotional behavioral deficits, including enhanced contextual fear and impaired fear extinction, and anxiety-like behavior, which are associated with reduced corticolimbic Allo levels and subsequent GABAergic neurotransmission dysfunction (Dong et al., 2001; Pinna et al., 2003, 2006b; Serra et al., 2006; Matsumoto et al., 2007; Zhang et al., 2014). Remarkably, patients with MDD and PTSD also show a CSF and brain Allo level down-regulation which is correlated with the severity of symptoms (Romeo et al., 1998; van Broekhoven and Verkes, 2003; Uzunova et al., 1998, 2006; Agis-Balboa et al., 2014). These findings suggest that the SI mouse may offer a suitable model to assess the effect of novel drugs to treat endophenotypic expressions of behavioral deficits that translate into symptoms of psychiatric disorders, including MDD and PTSD. Reduced corticolimbic Allo levels can be upregulated in humans by treatment with SSRIs that also correlates with improved symptoms (Romeo et al., 1998; Uzunova et al., 2006; Agis-Balboa et al., 2014). Importantly, patients who fail to show behavioral improvements also fail to show CSF Allo level upregulation (Uzunova et al., 1998, 2006). In SI mice, a single low dose of *S*-FLX, which is devoid of serotoninergic effects, by acting as a SBSSs, i.e., selectively stimulating brain Allo biosynthesis, is associated with reduced aggression and improvement of other emotional behaviors (Pinna et al., 2003, 2009; Pibiri et al., 2008; Nin et al., 2011a). This suggests that Allo analogs may offer a valuable treatment option. Importantly, BR297 was a strong anti-aggressive agent and the non-response rate of early adolescent SI mice that were treated with BR351 or BR297 was significantly lower than those receiving the higher dose of *S*-FLX. The other Allo’ analog tested, GNX was also efficient in reducing aggression in early SI mice and these results confirmed those of a previous study in which GNX also reduced anxiety-like behavior and fear responses (Pinna and Rasmusson, 2014). This is the first report showing that the novel Allo’ analogs, BR297 and BR351 show an anti-aggressive action in SI mice. Of note, the endocannabinoid PEA (15–20 mg/kg) was the only drug studied that improved aggressive behavior in all of the early adolescent SI mice. Collectively, these results provide further support for the evidence that neurosteroidogenic drugs or neurosteroid analogs are useful anti-aggressive agents and may provide benefits in treating a number of neuropsychiatric disorders characterized by impulsive aggression.

Clinical findings show that only 50% of depressed patients respond to first-line therapy antidepressants, while more than one third of responders develop resistance to antidepressants (Kemp et al., 2008). It is generally accepted that early traumatic experiences cause a poor response to SSRI treatments later in adulthood, representing one of main causes for pharmaco-resistance. For example, abuse in general but -most notably- abuse occurring at 7 years of age or younger predicted a lower response to 8 weeks of SSRI antidepressants (Williams et al., 2016). Likewise results from our study show that behavioral deficits are harder to improve in mice subjected to early social isolation than mice who are socially isolated later in adolescence using the same drug doses. Furthermore, while administering low steroidogenic doses of *S*-FLX to mice resulted in a lower response rate than other drugs tested, it resulted in a generally higher response rate than that observed when an SSRI is given to patients. Thus, one translational limitation of the present study as far as the clinical aspects of SSRI-resistance is concerned, is that we used low doses of *S*-FLX, banking on the neurosteroidogenic effects of this compound rather than administering higher doses that involve serotonergic mechanism and that better reflect doses administered in patients. Therefore, the high pharmacological resistance to SSRI at the high doses that target the serotonergic mechanism may be not observed or addressed by the current study design. Furthermore, these preclinical findings together with clinical observations of high incidence of resistance to current prescribed SSRI medication at supposedly steroidogenic doses suggest that the deficits in the activity of enzymes that are involved in Allo biosynthesis may not be fully counteracted by SSRIs in a portion of depressed and PTSD patients. However, it is also true that SSRIs at “high” serotonergic doses (as those administered in patients) remain to be thoroughly investigated for their ability to induce the Allo biosynthesis that they do at 1/10 of the doses required to engage serotonergic mechanisms. This hypothesis should be carefully examined in rodent stress models characterized by an Allo level downregulation. There are a number of additional speculations that can be drawn for the low pharmacological response rate of SSRIs in patients. For example, too high doses of SSRIs may not be required or necessary to improve symptoms and an excess of midbrain serotonin levels may even impair serotonin-regulated neuroplasticity or a high SSRI dosage may also masks beneficial drug effects by inducing severe side effects such as agitation, mental confusion, tremors, and seizures (Volpi-Abadie et al., 2013; Coplan et al., 2014; Ferri, 2016). These considerations call for clinical trials to assess whether low SSRIs doses that act as SBSSs would result in higher pharmacological response rate over the current large SSRI doses administered for PTSD and depression.

Our results showed that an alternative and valid strategy to overcome behavioral deficits might be to directly modulate GABA_A_ receptors with analogs of Allo. GNX, BR351, or BR297 may be a suitable future approach for patients for whom an SSRI/SBSS is ineffective because of their inability to overcome impairment in neurosteroidogenesis (Gulinello et al., 2003). In support of this hypothesis, previous studies conducted in our laboratory showed that a single dose administration with GNX given to SI mice blocked reconsolidation of fear memories and reduced contextual fear and facilitated fear extinction, which failed to re-emerge via “spontaneous recovery” (Pinna and Rasmusson, 2014). Reinforcing the impact of a neurosteroid-based therapy for PTSD and depression are recent studies showing that remission of post-partum depression was induced in 70% of patients treated with a two-day course of intravenous Allo compared to 10% who received placebo. Remarkably, this symptom improvement was rapid (60 h) and lasted for 30 days (Herper, 2016; Meltzer-Brody, 2016; Kanes et al., 2017).

Recently, the importance of the endocannabinoid system as a new target for stimulating neurosteroidogenesis has emerged. Direct activation of cannabinoid receptor type 1 (CB1) by Δ9-tetrahydrocannabinol (THC), the principal psychotropic compound of *cannabis sativa*, increased by 30-fold the synthesis of pregnenolone, the precursor of Allo (Vallée et al., 2014; Vallée, 2016). More evidence suggests that the endocannabinoid and neurosteroid systems are interrelated. For example, recent studies showed that PEA induces Allo biosynthesis in cultured astrocytes, and spinal cord and it regulates pentobarbital induced sedation by acting at the intracellular peroxisome proliferator-activated receptor (PPAR)-α (Sasso et al., 2012). Moreover, PEA is also able to induce antidepressant effects similar to those of fluoxetine (Yu et al., 2011). Studies in our laboratory show that PEA induces Allo biosynthesis in corticolimbic areas and this enhancement of neurosteroid levels is involved in the improvement of behavioral dysfunctions of SI mice (Locci and Pinna, 2017b). Therefore, drugs capable of inducing neurosteroidogenesis by targeting the endocannabinoid system could represent another important therapeutic alternative to treat depression, PTSD and anxiety disorders in SSRI non-responders.

## Conclusion

PTSD and depression are multifactorial disorders with different symptom clusters and involve neurochemical deficits that may vary among individuals. Current treatment relies on SSRIs, which are efficacious only in a portion of patients. Early life traumatic events are associated with poor response to SSRI treatment. To assess treatment suitability, it would be important to screen PTSD and depressed patients for neurosteroid level downregulation (e.g., Allo) as a biomarker for diagnostic assessment. This may be important to guide precision therapy and could be helpful for designing appropriate intervention with neurosteroid-based synthetic molecules, including analogs of Allo or drugs that stimulate its synthesis such as the cannabinoids.

Collectively, data reported in this paper offer preclinical evidence in support of treatment alternatives for SSRI non-responders. Future studies are necessary to further clarify the precise molecular mechanisms involved in their behavioral improvement.

## Author Contributions

AL performed the behavioral experiments, data analyses and graphic art work. GP designed the experiments, participated in data analyses and wrote the manuscript with AL. A-GM-N initiated the collaborative work with GP and contributed to the conception and design of the works aiming to compare the effects of neurosteroids analogs BR297 and BR351 to Ganaxalone. PG and MM synthesized BR297 and BR351 for evaluating the biological properties described in this article.

## Conflict of Interest Statement

A-GM-N,PG, and MM are the co-owners of patent number WO 2012127176 A1 and US 2014/0058079 A1. The other authors declare that the research was conducted in the absence of any commercial or financial relationships that could be construed as a potential conflict of interest.

## Footnotes

1 www.cohort.com
